# Lipid profile abnormalities seen in T2DM patients in primary healthcare in Turkey: a cross-sectional study

**DOI:** 10.1186/1476-511X-13-183

**Published:** 2014-12-06

**Authors:** Aclan Ozder

**Affiliations:** Medical Faculty, Department of Family Medicine, Bezmialem Vakif University, Adnan Menderes Boulevard, 34093 Fatih, Istanbul, Turkey

**Keywords:** Dyslipidemia, Glucose intolerance, Insulin resistance, Type 2 diabetes, Lifestyle modification

## Abstract

**Background:**

Diabetes is characterized by chronic hyperglycemia and disturbances of carbohydrate, lipid and protein metabolism. We aimed to research association between serum lipid profile and blood glucose, hypothesizing that early detection and treatment of lipid abnormalities can minimize the risk for atherogenic cardiovascular disorder and cerebrovascular accident in patients with type 2 diabetes mellitus.

**Methods:**

Fasting blood glucose (FBG), total cholesterol (TC), high density lipoprotein (HDL), low density lipoprotein (LDL), triglyceride (TG) and glycated haemoglobin (HbA1c) levels were evaluated. A hepatic ultrasound was performed for every diabetic to evaluate hepatosteatosis. The study was done from January 2014 to June 2014 among 132 patients with T2DM who were admitted to outpatient clinic of Family Medicine department in a university hospital. The patients whose taking multi-vitamin supplementation or having hepatic, renal or metabolic bone disorders (including parathyroid related problems) were excluded from the study for the reason that those conditions might affect the carbohydrate and lipid metabolism in diabetes. Test of significance was calculated by unpaired student’s t test between cases and controls. Correlation studies (Pearson’s correlation) were performed between the variables of blood glucose and serum lipid profile. Significance was set at p<0.05.

**Results:**

Results of serum lipid profile showed that the mean values for TC, TG, HDL and LDL in female patients were 227.6 ± 57.7 mg/dl, 221.6 ± 101.1 mg/dl, 31.5 ± 6.7 mg/dl and 136.5 ± 43.7 mg/dl, respectively. The mean values for TC, TG, HDL and LDL in male patients were 219.1 ± 34.7 mg/dl, 250.0 ± 100.7 mg/dl, 30.2 ± 7.4 mg/dl and 125.7 ± 21.4 mg/dl, respectively. Significantly higher mean serum levels of TC, TG and LDL and significantly lower mean serum levels of HDL were noted in patients with diabetes (p<0.001). FBG showed significant positive correlation with TC (p<0.05) and TG (p<0.05). Significant correlations were observed between serum levels of TC, TG, LDL and hepatosteatosis and HbA1c (p<0.05).

**Conclusions:**

The study showed widespread lipid abnormalities in the course of diabetes triggered dyslipidemia as hypercholesterolemia, hypertriglyceridemia, elevated LDL and decreased HDL. This study proposes the predominance of hyperlipidemia over increased prevalence of diabetic dyslipidemia.

## Background

Diabetes mellitus is a common metabolic disorder characterized by absolute or relative deficiencies in insulin secretion and/or insulin action associated with chronic hyperglycemia and disturbances of carbohydrate, lipid and protein metabolism [1]. Several previous studies have attempted to correlate blood glucose levels with serum lipid profile parameters [2, 3]. Research findings show that mainly body fat is responsible for increase in prevalence of this disease among the body composition components [1, 4, 5]. As early as 1988, it was described a multifactorial metabolic abnormality consisting of insulin resistance with compensatory hyperinsulinaemia, type 2 diabetes mellitus (T2DM), essential hypertension and hypercholesterolaemia [6, 7]. Today, however, the World Health Organization (WHO) and International Diabetes Federation (IDF) use the term “Metabolic Syndrome” to describe this clustering of conditions [8]. The term diabetic dyslipidemia comprises a triad of raised triglycerides, reduced high density lipoprotein (HDL) and excess of small, dense low density lipoprotein (LDL) particles. The lipid abnormalities are prevalent in diabetes mellitus because insulin resistance or deficiency affects key enzymes and pathways in lipid metabolism [9]. Micro-vascular and macro-vascular complications, including cardiovascular disease (CVD), retinopathy, nephropathy, and neuropathy, occur due to chronic uncontrolled hyperglycemia in diabetics [10, 11]. It has been proposed that the composition of lipid particles in diabetic dyslipidemia is more atherogenic than other types of dyslipidemia [12]. The causal association between atherosclerosis and dyslipidemia is well established. In diabetes the associated hyperglycemia, obesity and insulin changes highly accelerate the progression to atherosclerosis [13, 14]. In a recent study, it was observed significant trends for rising risk of coronary heart disease, stroke and all-cause mortality in relation to higher levels of baseline HbA1c in more than 11,000 participants in the Atherosclerosis Risk in Communities Study. For HbA1c categories of <6.5% and ≥ 6.5%, there was a significant association between fasting blood glucose levels and coronary heart disease, stroke or death from any cause [15]. It was attempted to correlate blood glucose levels with serum lipid profile parameters in previous studies [2] and it is clear that HbA1c values are lower in individuals with a decreased risk of micro-vascular complications [15].

In the present study, we aimed to research association between serum lipid profile and blood glucose, hypothesizing that early detection and treatment of lipid abnormalities can minimize the risk for atherogenic cardiovascular disorder and cerebrovascular accident in patients with type 2 diabetes mellitus.

## Results

We evaluated clinical and laboratory findings in 132 patients with T2DM. Of the total number of subjects, 61.4% (81) were females, and 38.6% (51) of the subjects were males (Table 1). The age range and mean age of female subjects was 37–75 years and 55.5 ± 9.2 while the age range and mean age for male subjects was 39–84 years and 54.8 ± 12.6 years, respectively. The mean BMI of the participants was 28.59 ± 3.25 kg/m^2^ (range: 26.10-44.98). Eleven out of 132 patients were obese in this study with BMI ranging from 31.02 to 44.98.

**Table 1:** **Gender distribution and age of patients with type 2 diabetes**

Gender	No. of patients	Age ^1^
Female	81	55.5 ± 9.2
Male	51	54.8 ± 12.6
Total	132	55.3 ± 10.5

^1^Units expressed as years.

Results of the blood glucose profile showed that all individuals were hyperglycaemic. The fasting blood glucose and HbA1c percentage among entire group was shown in Table 2.

**Table 2:** **The concentration of fasting blood glucose and HbA1c percentage in diabetic patients**

	Normal range	Study group range	Mean
Glucose (FBG)^1^			
Female	70 – 100	109–337	196.7 ± 61.2^2^
Male	70 – 100	135–339	217.1 ± 57.5^2^
HbA1c^3^			
Female	<6.5	6.5-11.30	8.46 ± 1.49^4^
Male	<6.5	6.5-11.40	8.84 ± 1.53^4^

^1^Units expressed as mg/dl.

^2^Statistically significant (p < 0.001) as compared to normal range for plasma blood glucose.

^3^Units expressed as percentage (%).

^4^Statistically significant (p < 0.001) as compared to normal range for HbA1c.

Results of serum lipid profile showed that there is no statistically significant difference between diabetic females and males as TC, TG, HDL and LDL compared (p > 0.05). However, a significant difference was found as compared to normal range for related serum lipids of diabetics (p < 0.001) (Table 3).

**Table 3:** **The results of TC, TG, HDL and LDL in serum of diabetic patients**

Lipid	Female			Male		
	Normal range	Study group	Mean	Normal range	Study group	Mean
TC^1^	<200	137-396	227.6 ± 57.7^2^	<200	143-292	219.1 ± 34.7^2^
TG^1^	<150	89-411	221.6 ± 101.1^2^	<150	90-432	250.0 ± 100.7^2^
HDL^1^	45-65	24-50	31.5 ± 6.7	45-65	20-42	30.2 ± 7.4
LDL^1^	<100	69-274	136.5 ± 43.7^2^	<100	83-161	125.7 ± 21.4^2^

^1^Units expressed as mg/dl.

^2^Statistically significant (p < 0.001) as compared to normal range for related serum lipids.

Mean value of the TG/HDL ratio which is considered a surrogate marker of insulin resistance was 7.98 ± 3.87 among entire group. In correlation studies, TG/HDL ratio showed significant positive correlation with HbA1c (p < 0.01). Among all patients, hypercholesterolemia was found in 93 (70.5%) individuals. Similarly, increased LDL cholesterol was found in 117 (88.6%) individuals. Of the participants only 39 (29.5%) were found to have normal values for TG. Grade I and II hepatosteatosis were found in nearly one third of diabetics and Grade III hepatosteatosis in 63 (47.7%) subjects while Grade IV hepatosteatosis was found in 27 (20.5%) of the study group by hepatic ultrasound (Table 4).

**Table 4:** **Distribution of hepatosteatosis grades among diabetics**

Hepatosteatosis	No. of patients	%
Grade I	21	15.9
Grade II	21	15.9
Grade III	63	47.7
Grade IV	27	20.5

In correlation studies, FBG showed significant positive correlation with cholesterol (p < 0.05) and TG (p < 0.05). The correlation of cholesterol with TG (p < 0.001) and LDL cholesterol (p < 0.001) were positive. However, HDL cholesterol showed a negative correlation with LDL cholesterol (p < 0.01). Findings were presented in Table 5.

**Table 5:** **Correlation studies between the blood glucose and serum lipid profile variables of diabetic patients**

	FBG	TC	TG	LDL	HDL	Hepatosteatosis	HbA1c
TG/HDL	0.577^2^	0.406^2^	0.518^2^	0.316^1^	−0.220^1^	0.793^2^	0.772^2^
FBG		0.331^1^	0.362^1^	0.213	0.061	0.773^3^	0.870^3^
TC			0.719^3^	0.870^3^	0.217	0.299^1^	0.373^1^
TG				0.377^1^	0.038	0.424^2^	0.371^1^
LDL					−0.235^2^	0.203	0.331^1^
HDL						−0.200	−0.043
Hepatosteatosis							0.901^3^

The values expressed as Pearson correlation co-efficients.

^1^Correlation is significant at the 0.05 level.

^2^Correlation is significant at the 0.01 level.

^3^Correlation is significant at the 0.001 level.

## Discussion

There were more females (61.4%) than males (38.6%) with T2DM in this study. The high proportion of females in this study may be due to the nature of population admitting to this hospital in that more of them seek medical attention than men under favour of having more free time because most of them were housewives.

In patients with diabetes, many studies have clearly established that complications are mainly due to chronic hyperglycemia that exerts its injurious to health effects through several mechanisms: dyslipidemia, platelet activation, and altered endothelial metabolism [16–18]. Both lipid profile and diabetes have been shown to be the important predictors for metabolic disturbances including dyslipidaemia, hypertension, cardiovascular diseases [19]. Lipids play a vital role in the pathogenesis of diabetes mellitus. Dyslipidemia as a metabolic abnormality is frequently associated with diabetes mellitus. Abnormalities in lipid metabolism have been reported in patients with diabetes mellitus accompanied by the risk of cardiovascular arteriosclerosis [20]. In the present study, significantly higher mean serum levels of total cholesterol, triglycerides and LDL cholesterol were noted in patients with diabetes, which are well known risk factors for cardiovascular diseases among patients, when compared to the normal values (Table 3).

In diabetes many factors may affect blood lipid levels, because of interrelationship between carbohydrates and lipid metabolism. Therefore, any disorder in carbohydrate metabolism leads to disorder in lipid metabolism and vice versa. Insulin resistance is a primary defect in the majority of patients with T2DM. In non-diabetic individuals insulin resistance in combination with hyperinsulinemia has a strong predictive value for future development for type 2 diabetes [21]. Several studies showed that insulin affects the liver apolipoprotein production and regulates the enzymatic activity of lipoprotein lipase and cholesterol ester transport protein, which causes dyslipidemia in diabetes mellitus. Moreover, insulin deficiency reduces the activity of hepatic lipase and several steps in the production of biologically active lipoprotein lipase [4, 22, 23]. Hypertriglyceridaemia usually accompanies decreased HDL cholesterol, which is also a prominent feature of plasma lipid abnormalities seen in individuals with diabetes [24, 25]. The cluster of lipid abnormalities associated with T2DM is defined by a high concentration of TG and small dense LDL and a low concentration of HDL cholesterol. The association between reduced HDL cholesterol levels and increased risk of heart disease is, on the other hand, well established, independently of TG levels and other risk factors [26, 27]. The possible mechanism responsible for hypertriglyceridaemia may be due to increased hepatic secretion of very low density lipoprotein (VLDL) and delayed clearance of triglyceride rich lipoproteins, which is predominantly due to increased levels of substrates for triglyceride production, free fatty acids and glucose [28]. In the present study, significant correlations were observed between serum levels of total cholesterol, triglycerides, LDL cholesterol and hepatosteatosis and blood concentrations of HbA1c (Table 5). Results of the correlation studies suggest a clear association between hyperglycemia and appearance of dyslipidemia. Our findings were in line with a previous study suggesting that the level of total cholesterol is usually normal or near normal if glycaemic control is adequate, and worsening of control raises the level [29]. Therefore, improving glycaemic control might substantially reduce the risk of cardiovascular events in diabetic patients.

Abnormal glucose reading is the commonest metabolic abnormality in people with T2DM accompanied by lower HDL levels, elevated LDL, hypercholesterolaemia, and hypertriglyceridaemia. Poor glycaemic control and hypertriglyceridaemia are significant biochemical abnormalities in patients with T2DM. Dyslipidemia management in people with diabetes mellitus, just like in any other individual, starts with a flawless evaluation that aims to identify secondary causes that might contribute to the abnormal lipid profile [30].

The triglyceride-to-HDL cholesterol (TG/HDL) ratio has been investigated recently for various potential clinical uses in adult and paediatric populations [31, 32]. Previous research has demonstrated its positive associations with adverse cardio-metabolic risk factor profiles, metabolic syndrome and prediction of incident diabetes or its complications [31, 32]. This may occur as the TG/HDL ratio demonstrates an association with insulin resistance [31–33]. A number of studies have shown that the TG/HDL ratio has a good correlation with the generally accepted methods to define insulin resistance and it is considered a surrogate marker of it [34]. The significant positive correlation of TG/HDL ratio with HbA1c found in this study is in line with the studies in the literature (p < 0.01) [31–34].

Although obesity and T2DM commonly co-exist [35], mean BMI in our study was in the overweight range. Subjects enrolled in the study were the patients who have diagnose of their diabetes for averagely 3 years. They were under control of a dietitian since the date of diagnose. This might be the cause that patients in our study were mostly overweight, but not obese.

Lifestyle changes, including increased physical activity and dietary modifications, are the milestones of management. There is need for increased efforts by family members, community and healthcare professionals towards preventive based approach in the management of diabetes. This can be achieved through increased emphasis on lifestyle modification strategies such as exercise, increased dietary restrictions and weight control strategies especially for those with impaired fasting glucose. Monitoring of lipid profile by blood tests done in regular intervals in primary healthcare also might play an important role to detect and take care of lipid abnormalities both in diabetics and non-diabetics. One of the strengths of this study is that it was the first study in Istanbul, Turkey that looked at association between serum lipid profile and blood glucose among patients with T2DM in primary healthcare. To the best of our knowledge, there has not been any previous study both performing blood tests and hepatic ultrasound on this group of subjects. Despite several important findings in the present study, relatively small sample size is considered as a limitation of it.

The highest priority for diabetic individuals who have poor glycaemic control should be to achieve almost near normal blood glucose levels, with the expectation that this effort will also cause an improvement in dyslipidemia [22, 36, 37]. Our study clearly shows that lipid profiles are abnormal in diabetes mellitus. Realizing that most of the diabetics have a high probability of developing cardiovascular and cerebrovascular disease, it is essential that an individual who is diabetic should take care of dyslipidemia. Our study definitely indicates the presence of hyperlipidemia which might be hazardous for diabetics.

## Conclusion

The present study suggested that common lipid abnormalities during diabetes induced dyslipidemia are hypercholesterolemia, hypertriglyceridemia and elevated LDL cholesterol. Results suggest a high prevalence of dyslipidemia, which might be playing a major role in the development of cardiovascular diseases and cerebrovascular accidents among diabetic patients. The optimal care for diabetic patients should include routine monitoring of blood glucose and serum lipid profile. Efforts to achieve lifestyle changes, such as weight reduction, physical exercise and smoking cessation should be encouraged and initiated first and then followed by medication with lipid lowering drugs prescribed in evidence-based necessary conditions. The optimum treatment with proper antidiabetic drugs to obtain fair glycaemic control should go concomitantly with lipid lowering drugs as well as with taken dietary precautions. When those mentioned above are done, we might properly speak about the benefits that primary healthcare could ensure to diabetics by the advantage of monitoring patients more closely.

## Methods

The cross-sectional study sample included Turkish participants diagnosed with T2DM. The study was done from January 2014 to June 2014 in the outpatient departments of Family Medicine clinic of the Bezmialem Vakif University Hospital in the largest city of Turkey, namely Istanbul, in the north-west region of the country and participating 132 subjects with T2DM (more than 6 months duration) were randomly selected and recruited in the study. The patients whose taking multi-vitamin supplementation or having hepatic, renal or metabolic bone disorders (including parathyroid related problems) were excluded from the study for the reason that those conditions might affect the carbohydrate and lipid metabolism in diabetes [38, 39]. Also those patients having history of malabsorption syndromes such as celiac disease or active malignancy or with active infection were excluded from the study. Written informed consent was taken from each subject, fulfilling the mentioned criteria at above, before study inclusion. Study subjects were asked to complete a generalized questionnaire that contains demographic information including past and present medical history, and to return after fasting for more than 8 hours for anthropometry and blood withdrawal. At the screening visit, blood samples were examined for levels of glucose and lipid profile. Subjects who had abnormal levels of these at bio-chemical laboratory tests were excluded.

Subjects were requested to visit the family medicine outpatient clinic following an overnight fasted state (≥8 hours) for anthropometry and blood withdrawal by the clinic nurse and physician on duty, respectively. Anthropometry included height (rounded to the nearest 0.5 cm), weight (rounded to the nearest 0.1 kg), and mean systolic and diastolic blood pressure (mmHg). Body mass index (BMI) was calculated as weight in kilograms divided by height in square meters. Fasting blood samples were collected and transferred immediately to a non-heparinized tube for centrifugation. Collected serum was then transferred to pre-labeled plain tubes and delivered to the bio-chemistry laboratory in Bezmialem Vakif University Hospital.

Fasting glucose, cholesterol and triglyceride were measured using an chemical auto-analyzer (Toshiba, Japan). Cholesterol esters will be hydrolyzed by cholesterol esterase. Cholesterol will be oxidized into cholest-4-en-3-on and hydrogen peroxide by bacterial cholesterol oxidase. Hydrogen peroxide in the presence of phenol and amino-4-antipyrine forms a complex of red color showing absorption maximum between 500–550 nm. It is based on the principle that triglycerides in the serum sample are hydrolyzed enzymatically by the action of lipase to glycerol and fatty acids. The glycerol formed is converted to glycerol phosphate by glycerol kinase (GK). Glycerol phosphate is then oxidized to dihydroxyacetone phosphate by glycerol phosphate oxidase (GPO). The librated hydrogen peroxide is detected by a chromogenic acceptor, chlorophenol-4-aminoantipyrine, in the presence of peroxidase (POD). The red quinone formed is proportional to the amount of triglycerides (TG) present in the sample and is measured at 546 nm. High density lipoprotein (HDL) is measured by using phosphotungstate precipitation method based on the principle that chylomicrons, very low density lipoprotein (VLDL) and low density lipoprotein (LDL) fractions in serum or plasma are separated from HDL by precipitating with phosphotungstic acid and magnesium chloride. After centrifugation the cholesterol in the HDL fraction which remains in the supernatant is assayed with enzymatic cholesterol method using cholesterol esterase, cholesterol oxidase, peroxidase and the chromogen 4-aminoantipyrine. The LDL cholesterol test is a two reagent homogenous system. The assay is comprised of two distinct phases. In phase one a unique detergent solubilizes cholesterol from non-LDL-lipoprotein particles. This cholesterol is consumed by cholesterol esterase, cholesterol oxidase, peroxidase and 4-aminoantipyrine to generate a colorless end product. In phase two a second detergent in reagent 2 releases cholesterol from the LDL lipoproteins. This cholesterol reacts with cholesterol esterase, cholesterol oxidase and a chromogen system to yield a blue color complex which can be measured bichromatically at 540/660 nm. The resulting increase in absorbance is directly proportional to the LDL concentration in the sample. The value of VLDL cholesterol was calculated as one-fifth of the concentration of triglycerides.

It should be noted that the Quality Assurance (QA) standards are maintained by TS EN ISO 15189, whereas the QA department audits the laboratory at regular intervals. Ethical approval was taken from the institutional review board (IRB) prior to the commencement of the study.

Ultrasound could be used contributively in detecting hepatosteatosis and it is a non-invasive and easily applicable method. However, liver biopsy remains the only accurate way to diagnose hepatosteatosis. There are 4 sonographic findings of diffuse fatty change in the liver characterising different grades: (1) a diffuse hyperechoic echotexture (bright liver), (2) increased liver echotexture compared with the kidneys, (3) vascular blurring, and (4) deep attenuation [40]. A hepatic ultrasound was performed for every diabetic in the study group in order to evaluate whether hepatosteatosis is present or not and, if present, to detect the grade of hepatosteatosis by a radiologist.

Data were analyzed using the Statistical Package for the Social Sciences version 16.0 (SPSS, Chicago, IL, USA). Normal continuous variables were presented as mean ± standard deviation. Test of significance was calculated by unpaired student’s t test between cases and controls. Correlation studies (Pearson’s correlation) were performed between the variables of blood glucose and serum lipid profile. Significance was set at p < 0.05.
